# Calcium Dysregulation in Alzheimer’s Disease: A Target for New Drug Development

**DOI:** 10.4172/2161-0460.1000374

**Published:** 2017-09-15

**Authors:** Yong Wang, Yun Shi, Huafeng Wei

**Affiliations:** 1 Department of Anesthesiology and Critical Care, Perelman School of Medicine, University of Pennsylvania, Philadelphia, PA 19104, USA; 2 Department of Anesthesiology, The First Affiliated Hospital of Guangzhou University of Chinese Medicine, Guangzhou 510405, China; 3 Department of Anesthesiology, Children’s Hospital of Fudan University, Shanghai, 201102, China

**Keywords:** Alzheimer’s disease, Calcium, Ryanodine receptor, Dantrolene

## Abstract

Alzheimer’s disease (AD) is a devastating neurodegenerative disorder and the most common cause of dementia among aged people whose population is rapidly increasing. AD not only seriously affects the patient’s physical health and quality of life, but also adds a heavy burden to the patient’s family and society. It is urgent to understand AD pathogenesis and develop the means of prevention and treatment. AD is a chronic devastating neurodegenerative disease without effective treatment. Current approaches for management focus on helping patients relieve or delay the symptoms of cognitive dysfunction. The calcium ion (Ca^2+^) is an important second messenger in the function and structure of nerve cell circuits in the brain such as neuronal growth, exocytosis, as well as in synaptic and cognitive function. Increasing numbers of studies suggested that disruption of intracellular Ca^2+^ homeostasis, especially the abnormal and excessive Ca^2+^ release from the endoplasmic reticulum (ER) via the ryanodine receptor (RYR), plays important roles in orchestrating the dynamic of the neuropathology of AD and associated memory loss, cognitive dysfunction. Dantrolene, a known antagonist of the RYR and a clinically available drug to treat malignant hyperthermia, can ameliorate the abnormal Ca^2^+ release from the RYR in AD and the subsequent pathogenesis, such as increased β-secretase and γ-secretase activities, production of Amyloid-β 42 (Aβ 42) and its oligomer, impaired autophagy, synapse dysfunction, and memory loss. However, more studies are needed to confirm the efficacy and safety repurposing dantrolene as a therapeutic drug in AD.

## Introduction

Alzheimer’s disease (AD) is a devastating neurodegenerative disorder and the most common cause of dementia among aged people [1,2]. AD may also result in abnormality in mood and personality [3,4]. Alzheimer’s disease is named after Dr. Alois Alzheimer, who first described this disease in his patient in 1906 [5]. AD represents one of the biggest diseases without effective treatment confronting human beings during this millennium [6]. Today, a new AD patient is diagnosed every 66 s in the United States. By 2050, one new case of AD is expected to develop every 33 s, resulting in nearly 1 million new cases per year [2]. In the US alone, an estimated 5.5 million patients are diagnosed with AD, a devastating neurodegenerative disease without effective treatment [4,7]. By 2050, the number of AD patient is expected to grow to 13.8 million [2,4]. Death usually occurs within 5 to 10 years after a clinical diagnosis [8]. The total estimated worldwide financial burden of dementia was $604 billion in 2010 [9]. AD not only seriously affects the patient’s physical health and quality of life, but also adds a heavy burden to their family and society. It is urgent to understand AD pathogenesis and develop the means of prevention and treatment. Considering the rapidly increased elderly population, AD has become a major health problem for human beings.

## AD Pathophysiology and Treatment Status

AD is a chronic devastating neurodegenerative disease without effective treatment [10]. Current approaches for management focus on helping patients slow or delay the symptoms of cognitive dysfunction [11,12]. There are many hypotheses about the pathogenesis of AD such as amyloid hypothesis, tau protein hypothesis, genetic hypothesis, excitatory amino acid hypothesis, chronic inflammation hypothesis, oxygen free radicals leading to neurodegenerative disease hypothesis, and neuronal apoptosis hypothesis. Amyloid cascade hypothesis is widely presumed to cause AD pathogenesis [13–15]. The autopsy of the pathological features is amyloid-β (Aβ) aggregates composed of senile plaques, intracellular neurofilament consisting of hyperphosphorylated tau protein deposits neurofibrillary tangles of tau (NFT) and loss of cerebral cortex caused by atrophy [16–18]. Many potential treatments for AD focused mainly on reducing levels of amyloid-β (Aβ) burden in the brain and inhibiting Aβ aggregation and promotion of Aβ clearance [19–21]. Despite the tremendous research looking into the molecular mechanisms of Aβ pathology, it is still unclear about the root causes of the AD related cognitive dysfunction [22,23]. Unfortunately, no drug of the amyloid-targeting the cascade is in the process to be approved for treatment of AD in patients [22,24]. Because Tau pathology play important roles in neurodegeneration, which is usually seen together with amyloid pathology, researchers also tried to develop new drugs targeting hyperphosphorylated NFT [22,25]. Studies have shown that there is a strong link between NFT deposition and neuronal loss related cognitive dysfunction [26–28]. Recent studies have suggested some tau genetic markers are associated with AD [29,30]. Unfortunately, there are no new drugs targeting tau pathology successful in patients up to now, although efforts are continued [22].

In order to block the progression of the disease in AD, we need to interfere with the pathogenic steps responsible for the clinical symptoms. Beside amyloid and tau pathology, alternative theories have been proposed for the pathogenesis of AD, such as inflammation, oxidative damage, iron deregulation, and cholesterol metabolism, etc. [31,32].

## Role of Calcium Signalling in Physiological Neural Processes and Dysregulation of Calcium Signalling in the Pathogenesis of AD

The calcium ion (Ca^2+^) is an important second messenger in the function and structure of nerve cell circuits in the brain. Ca^2+^ signalling regulates multiple neuronal functions, such as neuronal growth, exocytosis, synaptic plasticity and cognitive function [33–35]. Therefore disturbances in Ca^2+^ homeostasis can affect the neuron normal function and structure. A number of studies have shown that disruption of intracellular Ca^2+^ homeostasis plays important roles in orchestrating dynamic of the neuropathology of AD and associated memory loss, cognitive dysfunction [36–41].

Studies show that Ca^2+^ level in those neurons close to amyloid deposits is higher than normal resting level [42]. The elevated resting Ca^2+^ environment cloud promotes mechanisms of negative plasticity [43]. The mechanisms are an increase in calcineurin (CaN) expression and activity by elevated intracellular level. CaN is a Ca^2+^ signalling protein activated calmodulin (CaM), which is sensitive to subtle rises in intracellular Ca2^+^ levels. When CaN is activated, it is able to activate additional phosphatases, such as PP1, which further induce the long-term depolarization (LTD) that erases memories [44,45]. With blinding of Ca^2+^/CaM, CaMKII holenzymes can be activated. CaMKII also plays an important role in synaptic plasticity and memory formation. T286-autophosphorylation of αCaMKII is impaired at synapses in AD using post-mortem analyses and studies. The T286-autophosphorylation of αCaMKII in the hippocampus rescues deficits in contextual memory formation [46]. Studies suggested that neurotrophin-induced enhancement of p(T286)-αCaMKII leads to rescue of Aβ-induced deficits in LTP at hippocampal synapses [47]. Further, CaMKII has also been suggested to be a tau kinase. Studies with AD brain find that αCaMKII expression in cells usually co-localises with tau mRNA or NFT [48–50]. So, CaMKII dysregulation may therefore be closely related with Alzheimer’s disease. Small dose of sAβ1-42 impaired Ca^2+^ clearance from presynaptic terminals and increased the basal Ca^2+^ concentration in cultured rat hippocampal neurons. This caused an increase in the phosphorylation of Ca^2+^/calmodulin-dependent protein kinase IV (CaMKIV) and its substrate synapsin, which markedly inhibited synaptic vesicle (SV) trafficking along axons between synapses. sAβ1-42 prevents neurons from forming new synapses or adjusting strength and activity among neighboring synapses [51]. CaMKIV is crucially involved in Ca^2+^ induced CREB phosphorylation. Neural activity dependent CaMKIV signalling in the neuronal nucleus plays an important role in the consolidation/retention of hippocampus-dependent long-term memory [52].

Researches demonstrated that hTau accumulation caused remarkable dephosphorylation of cAMP response element binding protein (CREB) in the nuclear fraction both *in vivo* and *in vitro* studies. Activity-dependent activation of the transcription factor CREB is at a central converging point of pathways and mechanisms activated during the processes of synaptic strengthening and memory formation, as CREB phosphorylation leads to transcription of memory-associated genes [53]. Disruption of these mechanisms in AD results in a reduction of CREB activation with accompanying memory impairment [54]. hTau accumulation impairs synapse and memory by CaN-mediated suppression of nuclear CaMKIV/CREB signalling [55].

Due to spatial and temporal patterns of amyloid deposition, which does not correlate very well with the clinical degree of dementia in Alzheimer disease, the amyloid hypothesis remains controversial. In contrast, cognitive decline correlates very well with synapse loss [56]. It is actually the occurrence of ‘negative’ lesions such as synaptic loss which precedes neuronal loss that best correlates with the advancement of cognitive decline. Several reports have noted the progressive loss of synaptic boutons and other synaptic elements in brains of patients with symptoms ranging from mild cognitive impairment (MCI) to early-mild AD [57,58]. *In vitro* studies have shown that Aβ oligomers can directly bind to synaptic sites [59] and reduce long-term potentiation (LTP) [60,61].

In early AD, mild cognitive impairment may be due to synaptic dysfunction with no widespread synaptic loss and neurodegeneration. Soluble Aβ oligomers can adversely affect synaptic structure and plasticity even at extremely low concentrations. In many cases, AD transgenic mice show abnormal synaptic transmission and impaired LTP usually before plaque formation [62,63]. Ca^2+^ is an essential mediator of basal synaptic transmission, short and long forms of synaptic plasticity, and dendritic spine morphology [64]. In AD mouse models at asymptomatic or early disease stages, the increased Ca^2+^ affects the synaptic pathophysiological processes by increasing both frequency of spontaneous synaptic potentials and negative plasticity [65,66].

Negative plasticity was proposed to explain cognitive decline in older people. Their framework describes a self-reinforcing, downward spiral of negative brain plasticity whereby declining brain function is attributable to a combination of disuse reduced quality of sensory-perceptual processing and weakened neuromodulatory control. In combination, these factors increase reliance on simplified cognitive processing at the expense of more complex processing capacity [67].

Additionally, Aβ plaque deposition was needed to induce calcium overload [42]. Aβ oligomers can increase cytosolic calcium through forming novel pores on plasma membranes and can stimulate mGluR5 which increases InsP3 production and Ca^2+^ release [68–71]. Recent studies showing that intracellular Aβ oligomers can stimulate G-protein-mediated Ca^2+^ release from the Endoplasmic reticulum (ER) through InsP3 [72]. The ER is a particularly intriguing organelle that actively removes Ca^2+^ from the cytoplasm and can release stored Ca^2+^ into cytosolic space through ER membrane calcium channel receptors, Inositol 1,4,5-Trisphosphate receptor (InsP3R) or the ryanodine receptor (RYR). Excessive Ca^2+^ release from the ER via activation of RYR and/or InsP3R is associated with amyloid and tau pathology and contributes to memory and learning loss in AD40 [73,74], while RYR can be activated by Ca^2+^ itself and may amplify the function of InsP3R via a calcium activated calcium release mechanism [75,76]. This may decrease or deplete Ca^2+^ levels in the ER. The abnormally low Ca^2+^ level will cause a decrease in vATPase production due to the protein-folding reaction depending on high concentrations of Ca^2+^ in the ER [77]. When vATPase maturation in the ER is disturbed, the proper pH value in lysosomes can’t be maintained due to decreased vATPase, which leads to impaired lysosomal acidification and function and subsequent autolysosome and autophagy function. It is interesting to note that ER Ca^2+^signaling abnormalities; plasticity and memory deficits precede detectable amyloid and tau pathology in AD [36].

ER is an important subcellular organelle for protein synthesis, modification and folding. ER stress and associated unfolded protein accumulation is triggered by the disruption of Ca^2+^ homeostasis. ER stress can stimulate cells to cope with unfolded protein responses, which promote protein folding or degradation of abnormal folding proteins [78]. Protein misfolding and aggregation are common pathogenic mechanisms in a number of human diseases, including AD. Perturbations of the function or integrity of the ER such as the accumulation of misfolded proteins in the ER lumen, results in a condition-termed ER stress. To avert this condition, cells activate an integrated array of adaptive intracellular signaling cascades known as the unfolded protein response (UPR). ER stress is induced during AD, and has been indirectly implicated as a mediator of Aβ neurotoxicity. In neurodegenerative diseases like AD, these abnormal reactions may play an important role [79]. ER stress could be the consequence of aberrant cellular signaling induced by the interaction of Aβ oligomers with membrane receptors, although these mechanisms are possible contributors to Aβ neuropathology.

Aβ42 expression induces strong ER stress response and the strongly activated UPR failure to buffer the misfolded protein load, leading to cellular dysfunction and a shorter chronological life span (CLS) [80]. Multiple studies have demonstrated that Aβ oligomers can activate PKR and induce ER stress by eliciting the TNF-α pathway [81,82]. Additionally, Aβ may stimulate ER Ca^2+^ release through ryanodine receptors and IP3 receptors, which triggers ER stress, neuronal apoptosis and mitochondrial fragmentation [72,83]. ER stress and hyperphosphorylated tau could be induced by each other in a cycle to propagate AD pathology [84]. Furthermore, studies have shown that mutations in PS1 inhibit ER stress-induced lREla PERK autophosphorylation and eIF2α phosphorylation in ER membranes. It has been suggested that familial AD-linked PS1 mutations suppress the activation of IRE-1α. This predisposes cells to become more susceptible to ER stress due, in part, to decreases in protein chaperone synthesis as a result of reduced UPR induction [85,86]. The aberrantly spliced isoform of PS2 (PS2V) is also linked to AD. Similar to the PS1 mutations, this isoform increases the vulnerability of the cell to ER stress [87].

The most abundant microtubule-associated protein is the Tau protein. In healthy brains, the combination of tau protein and tubulin promotes its polymerization to form microtubulins. Tau proteins then combine with microtubulins to maintain microtubule stability and induce microtubules into bundles. However, tau protein in the brain of AD patients is abnormally hyper phosphorylated, which leads to biological function loss [88]. Temporarily increased intracellular calcium signaling would induce prolonged increased tau phosphorylation via glycogen synthase kinase 3-β (GSK-3β) pathway in human neuroblastoma SH-SY5Y cells [89]. On the other hand, when the hippocampal and cortical neurons were cultured with tau protein, significantly increased intracellular calcium through muscarinic receptor was observed [90]. The cytoplasmic protein tau normally serves to stabilize microtubules which form ‘tracks’ that facilitate intracellular vesicle trafficking and axonal elongation and maturation. This is highlighted by the finding that knocking down tau leads to severe neurite growth defects in primary cerebellar neurons [91]. However, certain insults cause an imbalance between the activities of tau kinases and phosphatases that lead to the abnormal phosphorylation of tau [92]. In its hyperphosphorylated state, tau becomes soluble and, in turn, polymerizes to form oligomers and/or NFTs [93].

Emerging evidence indicate that many calcium-related proteins are involved in the phosphorylation of tau. In vivo experiment CaKMII-α and hyper phosphorylated tau protein in hippocampus slices using double-labeling immunofluorescence methods, indicats that CaKMII-α might be involved in tau phosphorylation [48]. In the meantime, an N-methyl-D-aspartate (NMDA) receptor antagonist has been clinically used as an effective symptomatic treatment. Another in vitro experiment further confirmed phosphorylation of tau protein that was catalyzed by phosphatidylserine and phophatidylethanolamine via CaKM, which was identified by sodium dodecyl sulfate-polyacrylaide gel electrophoresis [94]. Calcium phosphatase calcineurin influenced tau metabolism. Reduced calcineurin activity would increase extracellular phosphorylated tau [95]. Similarly, the calcium-induced phosphorylation of tau mediated by glycogen synthase kinase 3 (GSK3) and cyclin-dependent kinase 5 (CDK5) could be dephosphorylated by calcineurins [96]. Meanwhile, increased activity of calpains regulated GSK3 and Cdk5 from the initial too late stages of the disease leads to hyperphosphorylated tau, synaptic degeneration and memory loss [97–99]. It was proposed that calpain inhibitor could be a novel treatment for the disease. Rao et al. reported CDK5 activation, tau hyperphosphorylation, and tau accumulation in brains of Tau P301L mice that were rescued when the mice were treated with selective calpain inhibitor [100].

The presenilin-1 (PS1) and Presenilin-2 (PS2) genes have been identified in AD pathogenic most related to early onset, autosomal dominant type [101]. Mutations in PS1 that cause early-onset inherited AD increased Ca^2^+ release through the ER InsP3R and RYR [102–104]. The number and function of RYRs are abnormally increased in different brain regions of AD mice and patients, which may exaggerate Ca^2+^ signalling in synaptic terminals and thereby render them vulnerable to dysfunction and degeneration in the settings of aging and amyloid accumulation in AD [105–107]. Recent studies suggested that mutated PS2 or amyloid precursor protein (APP) also contributed to the calcium dysregulation and pathogenesis of AD by over activation of RYR37 [104,108–110]. Obviously, the ryanodine receptor over activation and abnormal Ca^2+^ release from the ER play important roles in AD pathogenesis and the adequate inhibition RYRs over activation may be a new therapeutic target for the treatment of AD.

**Figure 1:**
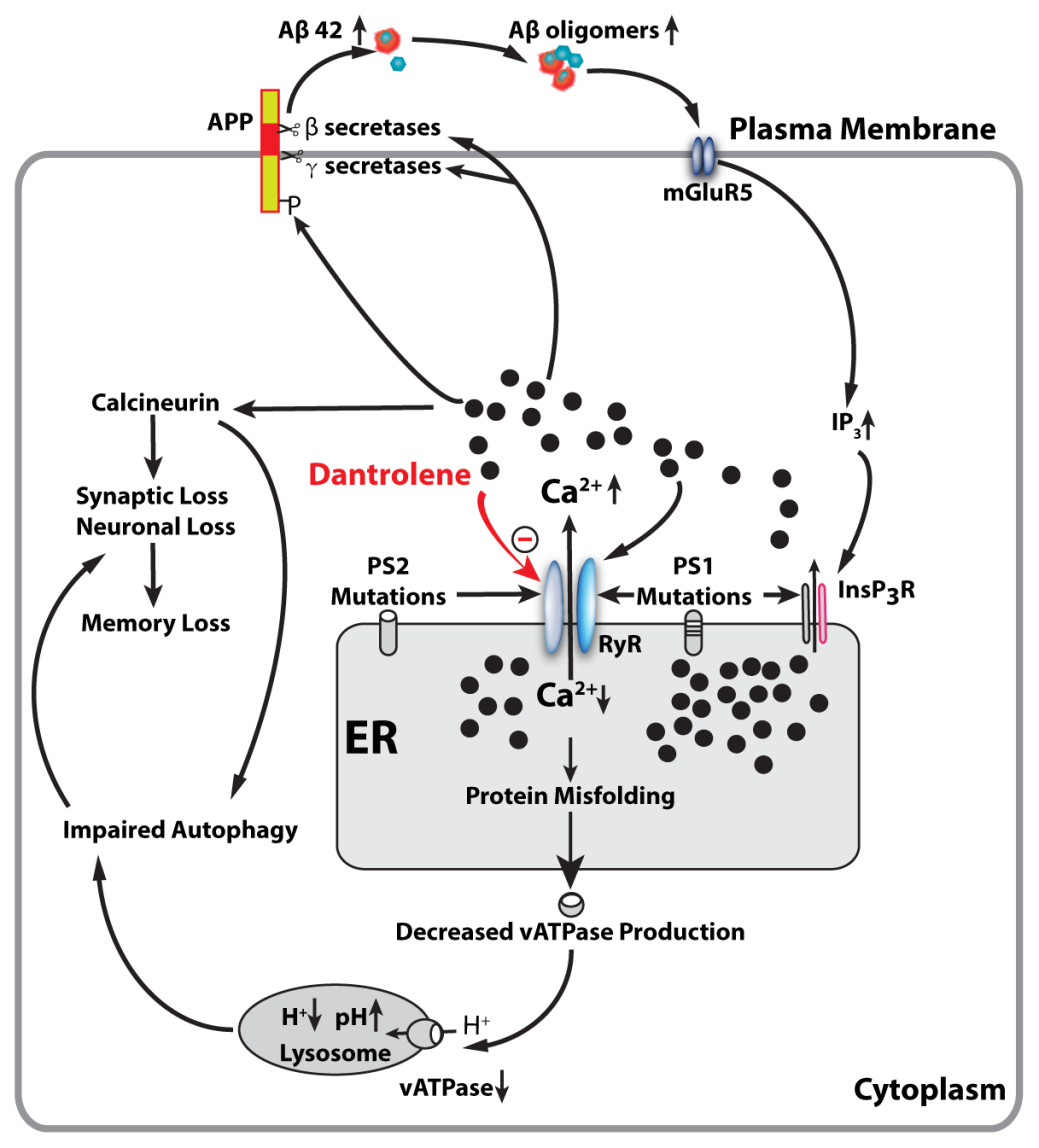
ER Ca^2+^ dysregulation in the pathogenesis of AD and the effect of dantrolene.

Dantrolene is a known antagonist of the RYR and is used clinically to treat malignant hyperthermia, muscle spasms and neuroleptic malignant syndrome. Dantrolene has been demonstrated to mitigate the amyloid pathology, synapse and memory loss in various AD tissue culture and animal models [73,75,111,112]. Therefore, dantrolene is theoretically a potential drug to reverse the calcium dysregulation and neuropathology in AD and restore cognitive dysfunction. In fact, our previous study has demonstrated that that long-term oral treatment with dantrolene in aged 3xTg-AD mice significantly decreased intraneuronal amyloid accumulation in the hippocampus. Studies show that dantrolene through the modulation of RyR-mediated Ca^2+^ release from ER and β- and γ-secretases activities leads to the reduction of Aβ production to prevent learning and memory decline [113]. It has been recently proposed that intraneuronal free oligomer of amyloid, rather than aggregated plaques, play important roles in synapse dysfunction and loss, as well as neurodegeneration [114–116]. The exact molecular mechanism of inhibitory effects of dantrolene on RYR is not clear, while recent studies suggested that certain cytosolic calcium concentration of magnesium ions are needed for effective RYR inhibition by dantrolene [117]. Overall, recent pilot studies suggested that calcium dysregulation in AD may be a potential therapeutic treatment for AD. Considering the earlier development of calcium dysregulation than amyloid pathology and importance of early treatment even before clinical symptoms, drugs targeting calcium dysregulation, such as dantrolene, may have good potential to facilitate the preclinical treatment in AD as possible effective therapeutic drugs (Figure 1).

Mutations in presenilin cause increased Ca^2+^ release from the ER through InsP3 (InsP3R) and ryanodine (RYR) two primary calcium channels. Abnormal elevation of cytosolic Ca^2+^ increase phosphoarylation of APP protein and activities of β- and γ-secretases, resulting in increased production of Aβ42 and Aβ oligomers, which in turn, further promote InsP3R-mediated Ca^2+^ release from ER by activating postsynaptic mGluR5 mediated InsP3 production. RYR can be activated by Ca^2+^ itself and therefore may function as an amplifier for Ca^2+^ release from the ER triggered by initial InsP3R activation. Abnormal decrease or depletion of ER Ca^2+^ level result in accumulation of misfolded proteins in the ER and decreased normal protein synthesis and secretion, including vATPase for lysosome, which then lead to decreased hydrogen concentration and elevated pH in lysosome. Dysfunctional lysosome lead to impaired function of autolysosome and overall autophagy function. On the other hand, the increased cytosolic Ca^2^+ activates calciuneurin which induces the synaptic loss and memory loss directly or via impaired autophagy. Dantrolene is a known antagonist of the RYR and inhibit excessive Ca^2+^ release from ER to cytosolic space and subsequent detrimental effects from abnormal elevation of cytosolic Ca^2+^ and depletion of ER Ca^2+^ in AD pathology.

## Future Strategy for New Drug Development

Alzheimer’s disease has shown insidious onset and a progressive dementia. It is a multifactorial complex disorder of the brain. So the treatment is equally complex and is a huge challenge. From a clinical perspective, interventions that target treatment AD through early disease diagnosis, combination therapies, lifestyle changes, stem cell therapy and exercise interventions show promise for brain health [32,118–121]. Studies have shown that if the treatment is performed before the diagnosis, the outcome is better. So, the hope is, treatments in the future should be initiated in its earliest stages, such as when calcium dysregulation start which is earlier than amyloid pathology, before occurrence of irreversible brain damage or mental decline. Research on new strategies for earlier diagnosis seems to be among the most advanced areas in AD research. An effective approach of detecting early calcium dysregulation in AD brain will help the effectivity of early treatment by drugs targeting calcium dysregulation pathology.

Several potential biomarkers are being studied for their ability to indicate early stages of Alzheimer’s disease. For examples, beta-amyloid and tau levels in cerebrospinal fluid and brain changes detectable by imaging. PET scan is one of these imaging technologies which utilize a radioactive tracer to look for pathological markers of the disease, and it has made it possible to isolate tau tangles in the brain. PET scan imaging is a relatively non-invasive detection method that may help with earlier diagnosis. Recent research shows that these markers may change at different stages of AD process [122,123].

Researchers are looking for new ways to treat Alzheimer’s. Current Alzheimer’s treatments temporarily help relieve the symptoms of memory loss and cognitive dysfunction with thinking and reasoning, but do not treat the underlying disease, and delay of its progression.

Future AD treatments may include a combination of medications, similar to the strategies of treatments for many cancers or HIV/AIDS. Dendritic spine defects clearly contribute to cognitive decline observed in AD. These defects are considered an early event in memory circuit’s destabilization and should be taken into account for future development of investigational drugs. Novel pharmacotherapies should not be limited to the postulates of the amyloid cascade hypothesis. Events occurring at the synapse may prove to be instrumental in understanding the underlying pathology of this devastating disease.
